# Host age is not a consistent predictor of microbial diversity in the coral *Porites lutea*

**DOI:** 10.1038/s41598-020-71117-4

**Published:** 2020-09-01

**Authors:** Benjamin J. Wainwright, Geoffrey L. Zahn, Lutfi Afiq-Rosli, Jani T. I. Tanzil, Danwei Huang

**Affiliations:** 1 Yale-NUS College, National University of Singapore, Singapore, 138527 Singapore; 2 Biology Department, Utah Valley University, 800 W. University Parkway, Orem, UT 84058 USA; 3 Department of Biological Sciences, National University of Singapore, 16 Science Drive 4, Singapore, 117558 Singapore; 4 Tropical Marine Science Institute, National University of Singapore, 18 Kent Ridge Road, Singapore, 119227 Singapore

**Keywords:** Ecology, Evolution, Microbiology, Molecular biology, Ecology, Environmental sciences, Ocean sciences

## Abstract

Corals harbour diverse microbial communities that can change in composition as the host grows in age and size. Larger and older colonies have been shown to host a higher diversity of microbial taxa and this has been suggested to be a consequence of their more numerous, complex and varied micro-niches available. However, the effects of host age on community structure and diversity of microbial associates remain equivocal in the few studies performed to date. To test this relationship more robustly, we use established techniques to accurately determine coral host age by quantifying annual skeletal banding patterns, and utilise high-throughput sequencing to comprehensively characterise the microbiome of the common reef-building coral, *Porites lutea*. Our results indicate no clear link between coral age and microbial diversity or richness. Different sites display distinct age-dependent diversity patterns, with more anthropogenically impacted reefs appearing to show a winnowing of microbial diversity with host age, possibly a consequence of corals adapting to degraded environments. Less impacted sites do not show a signature of winnowing, and we observe increases in microbial richness and diversity as the host ages. Furthermore, we demonstrate that corals of a similar age from the same reef can show very different microbial richness and diversity.

## Introduction

Corals contain diverse and complex microbial communities that play critical roles in maintaining and promoting host fitness and survival^1–3^. They are involved in nutrient cycling^4^, and help prevent colonization by pathogenic microbes through the occupation of potential niches^5^ and the production of specific antibacterial compounds^2^. These communities can differ over space and time, among host species, and with environmental perturbations^6–9^. Yet, some coral-associated bacterial communities remain constant with no discernible temporal changes^10,11^. These contrasting patterns demonstrate some of the challenges faced when attempting to understand the coral microbiome.

Numerous studies show that host-associated microbes can and do differ over comparatively small spatial scales^7,12–14^. However, few have explicitly examined how host age affects the microbial community, and those that have are limited by colony size as a proxy to obtain a crude estimate of age^15,16^. These studies show conflicting patterns with one reporting a steady, yet significant decrease in bacterial diversity with increasing size^16^, while the other demonstrates a general stepwise increase in bacterial diversity with increasing size followed by a slight decrease in diversity of the largest and assumed oldest corals^15^. However, it should be noted that the latter study by Williams et al.^15^ is limited by the young age of the assumed oldest corals, which have been estimated at only 10–12 years old. Pollock et al.^16^ suggest that early coral recruits and larvae have more diverse microbiomes in comparison to later life stages, which is consistent with previous work suggesting that a ‘winnowing’ of the microbial assemblage takes place with increasing host age. In other words, the microbiome slowly adjusts over time until it is fine-tuned and adapted to local environmental conditions^17^. Conversely, the pattern of increasing microbial diversity with host age reported by Williams et al.^14^ is thought to follow a successional process similar to patterns described within the human gut microbiome, specifically that diversity increases with age^14,18,19^. For a coral, this pattern could be a consequence of developmental changes that occur as it grows from a small larva to a large adult colony, so that larger and older hosts are associated with more numerous, complex and varied niches that can support more microbial species^15^. A similar phenomenon is observed in birds, where bacterial diversity increases with time from birth^19^. It is important to note that these previous attempts to investigate the relationship between coral age and microbiomes have varied in terms of the range of coral ages studied and the methods used to estimate age and microbial community diversity. Therefore, we seek to develop a more controlled test of this relationship by directly observing a wide range of accurately-aged coral samples (over 93 year) and by next-generation sequencing of bacterial 16S amplicons.

The reef-building coral *Porites lutea* is one of the most widespread and abundant corals throughout Southeast Asia^20–22^. This coral shows a massive growth form that is characteristically boulder shaped, the colony grows via linear extension showing observable ‘tree ring’-like growth patterns that can be used to generate accurate and relatively easy estimates of age^23^. These features make it especially useful as a model system to investigate potential influences of coral age on microbial diversity.

Here, we aim to quantify the effects of *P. lutea* host age on the diversity, richness and structure of the associated microbial community*.* For the first time in studies of this nature, coral host age was determined using established and accurate methodology rather than using size as a proxy. Furthermore, we comprehensively characterise the bacterial communities associated with *P. lutea* throughout Singapore and examine the extent to which these communities vary spatially. More broadly, our results serve as a baseline allowing us to track temporal changes in the microbiome of *P. lutea*, a ubiquitous and important member of Indo-Pacific reef communities.

## Results

Using high-throughput sequencing, we characterised the bacterial communities associated with 160 *Porites lutea* colonies collected from eight sampling sites in Singapore. A total of 14,374,065 sequences were generated on the Illumina MiSeq platform, and after filtering to remove chimeric sequences and any sequences that did not pass quality control a total of 12,582,075 sequences were retained for further analysis (for sequencing statistics of each sample, see Supplementary Table S1). Rarefaction curves showed that the asymptote of the number of amplicon sequence variants (ASVs; unique 16S rRNA gene sequences) was reached for each sample, indicating sufficient sequencing depth was achieved; ASV richness was comparable to previous work in the region^1,10,24^ (Supplementary Fig. S1). Of the 160 samples, we successfully determined the age of 91 individual colonies; logistical reasons prevented the coring of all 160 colonies. Corals ranged in age between 10 and 103 year, with the majority (71%) 21–50 year (Supplementary Table S2).

**Figure 1:**
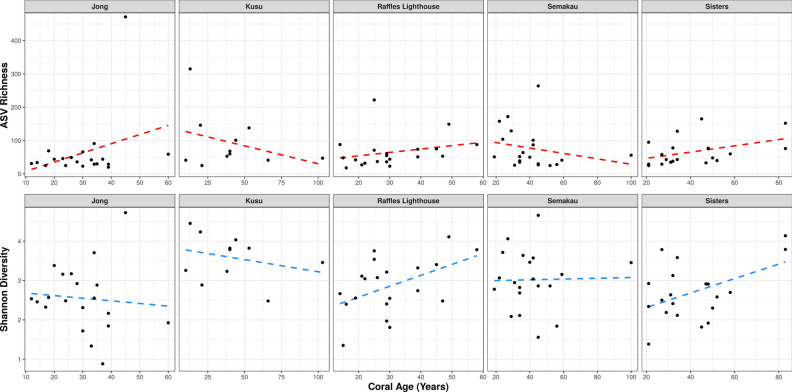
Relationship of bacterial diversity (ASV richness and Shannon diversity) against coral age, with each point representing a single colony. Fitted predictor lines determined by the optimal generalised linear model (ASV richness and Shannon diversity as functions of coral age and site, including the interaction term). See Supplementary Table S4 for a summary of the generalised linear models.

Using the optimal generalised linear models as determined by Akaike information criterion (AIC) (Supplementary Table S3)—Diversity ~ CoralAge * Location, and Richness ~ CoralAge * Location—we find site-dependent patterns between host age and both bacterial ASV richness and diversity. Samples collected from the islands of Kusu and Semakau both show significant predicted decreases in richness with increasing host age (*P* = 0.01 and *P* = 0.03, respectively) (Fig. 1, Supplementary Table S4). Jong, Raffles Lighthouse and Sisters all show predicted increases in richness that are not significant. Three of the islands (Raffles Lighthouse, Sisters and Semakau) all show predicted increases in bacterial diversity with increasing colony age, while Jong and Kusu show predicted decreases in diversity, though none of these are significant (Fig. 1, Supplementary Table S4). In all cases, corals of similar ages can display very different levels of diversity and richness. For example, ten corals aged 21–30 year at Raffles Lighthouse have Shannon diversity values of 1.7–3.7 (Fig. 1, Supplementary Fig. S2). In general, ASV richness follows the same trend as Shannon diversity (i.e., when ASV richness increases, Shannon diversity also increases), except at Jong where ASV richness increases as diversity decreases.

**Figure 2:**
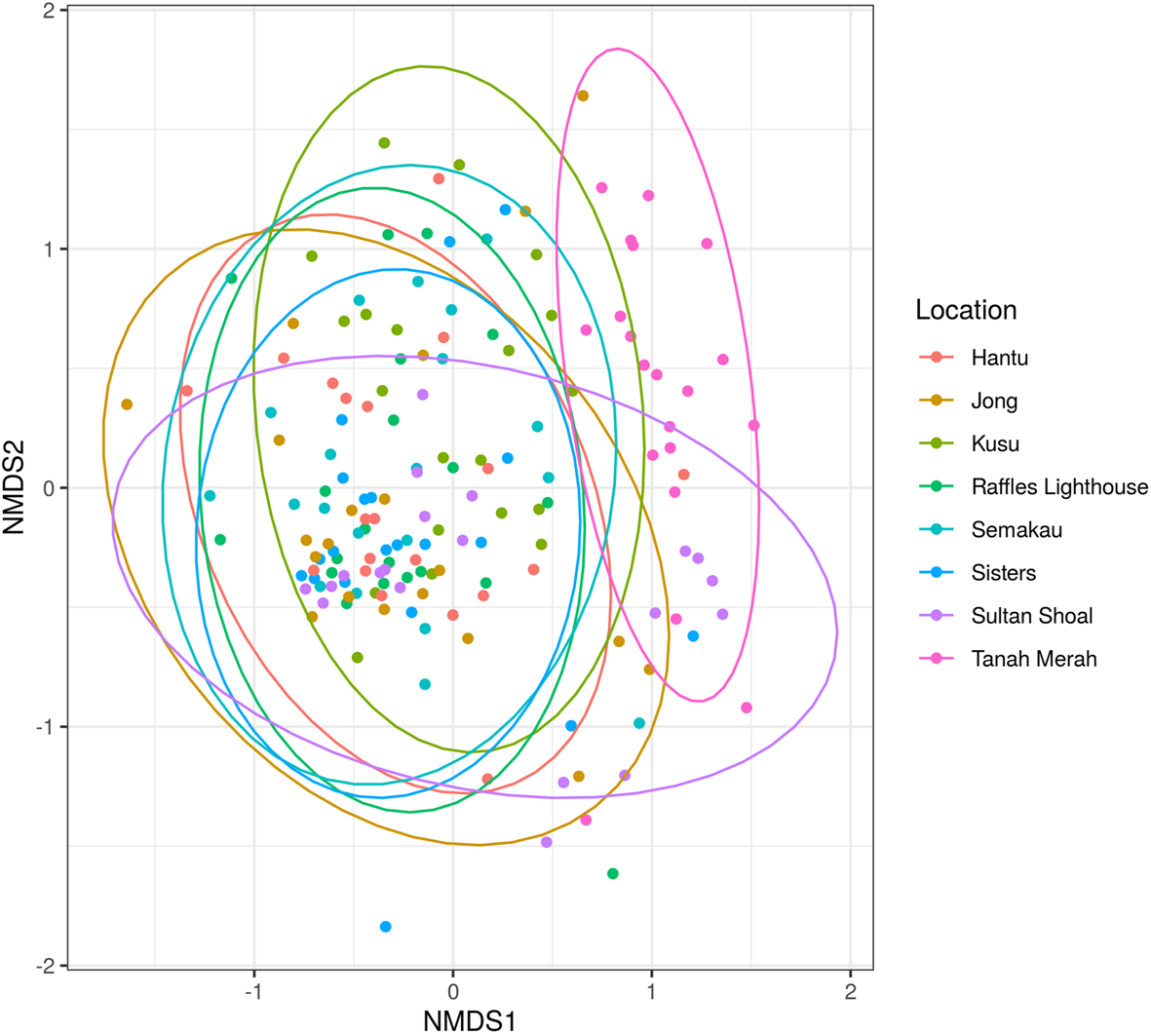
Non-metric multidimensional scaling (NMDS) of bacterial communities based on Bray–Curtis dissimilarity, coloured by location. Stress = 0.244, Non-metric fit, *R*^2^ = 0.94.

Permutational multivariate analysis of variance (PERMANOVA) performed on aged corals indicates that sampling location is a significant factor determining bacterial community structure (*R*^2^ = 0.068; *P* = 0.004), while coral age is not significant (*R*^2^ = 0.011; *P* = 0.438) (Supplementary Table S5, Fig. S3). Location remains a significant factor when all 160 samples are analysed (*R*^2^ = 0.118; *P* = 0.001) (Supplementary Table S5). Non-metric multi-dimensional scaling (NMDS) suggests that samples collected from Tanah Merah are more similar to one another in microbial community structure than all other locations (Fig. 2), and this uniqueness is supported by the network plot showing Tanah Merah as a distinct group that shares more ASVs with itself than other sampled locations (Supplementary Fig. S4).

**Figure 3:**
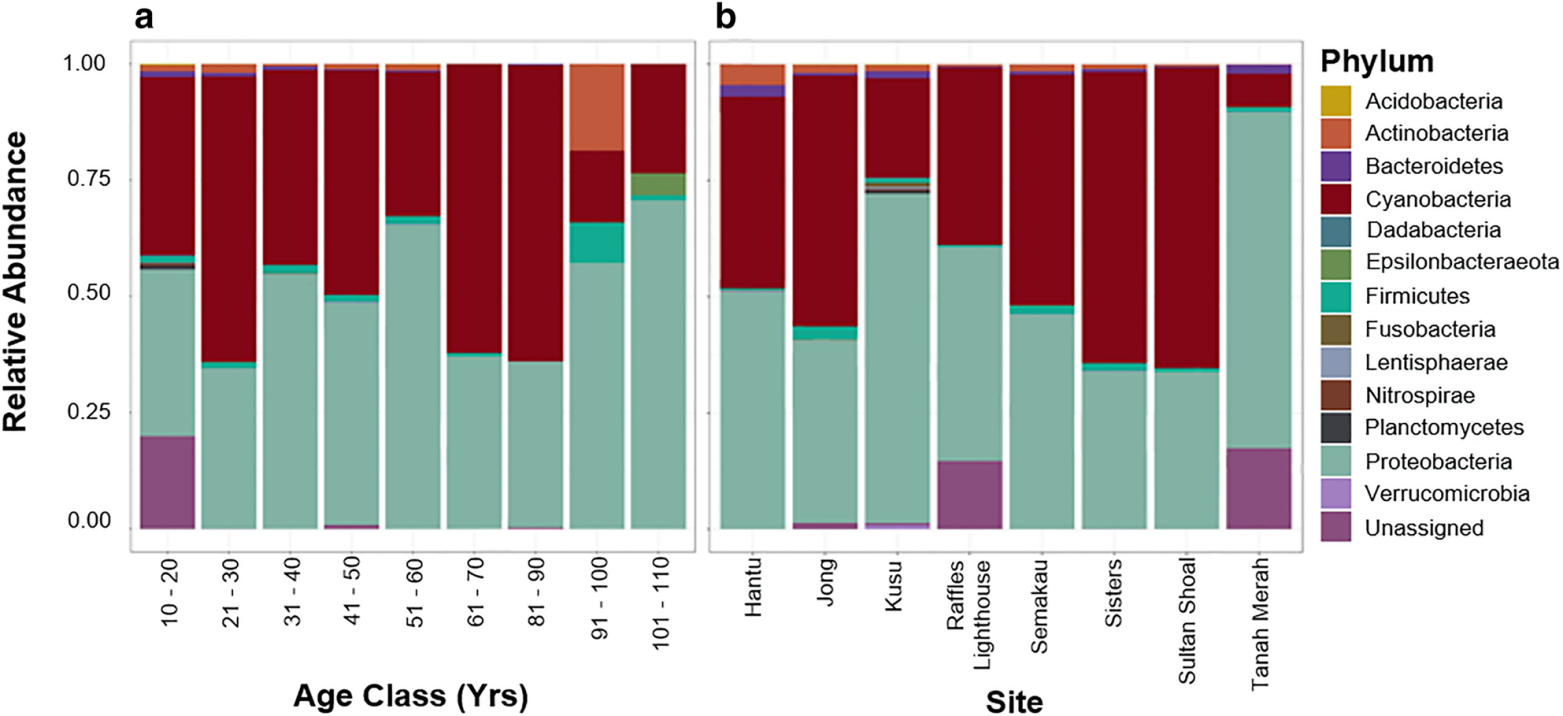
Stacked bar plots of relative bacterial abundance: (**a**) for samples in each age class. There are no corals in the 71–80 year age class, with only 91 corals aged in total; and (**b**) bacterial abundance for each sample site (all 160 corals).

Samples collected from all locations and age classes are dominated by Cyanobacteria and Proteobacteria (Fig. 3). The relative abundances of both of these phyla are generally consistent throughout the first 90 year of the corals’ life. The limited sampling of corals in the 91–100 year and 101–110 year age groups (*n* = 2) requires care in the interpretation of bacterial diversity. However, in both corals older than 90 year, the proportion of Cyanobacteria relative to Proteobacteria is the lowest among all age classes. The single 91–100 year coral has the highest proportions of Actinobacteria and Firmicutes, while the sample from the oldest age category, 101–110 yr, has the highest proportion of Epsilonbacteraeota (Fig. 3). At the class level, all samples across all age groups are dominated by Gammaproteobacteria. No one bacterial order, family, or genus appears to be dominant in any age class (Supplementary Figs. S5–S7). Three families—Moraxellaceae, Pseudomonadaceae and the Burkholderiaceae—are differentially abundant in corals of different age classes based on beta-binomial regression models, but these taxa are not specifically associated with older or younger corals, effect sizes are small, and there are no significant differences in bacterial dispersion for any taxa (Supplementary Table S6).

## Discussion

This work documents the complexities involved in assessing how host age influences microbial diversity and richness. Some localities show positive patterns in diversity and richness as age increases, while others show the opposite trend. It is likely that we are seeing evidence of both previously described hypotheses^15,16^. For example, winnowing of the microbiome appears to be occurring in some corals as they become adapted to specific local environmental regimes^17^, while the positive age-dependent diversity could be a result of increases in the age and size of host along with the corresponding increase in microbial niches.

Numerous studies have documented reduced bacterial diversity in degraded environments or in stressed hosts^15,25–29^, and the more stressful environmental conditions faced by corals at Jong and Kusu could be responsible for the observed microbial winnowing. In support of this idea, these sites are freely accessible to the public, whereas access to the reefs where we see the clearest increases in diversity (Raffles Lighthouse and Sisters) is regulated. In particular, Raffles Lighthouse is off limits to recreational and commercial uses, and research access is strictly controlled by the Maritime and Port Authority of Singapore. It is also the farthest from the influence of the urbanised mainland and consistently has the highest hard coral cover (> 50% of the benthos) among sites over several decades^30–32^, indicating an environment where stress is low in comparison to other reefs in Singapore. Correspondingly we see a clear pattern of increasing bacterial diversity with increasing host age here. Similarly, use of the waters surrounding the Sisters Islands, part of Singapore’s first and only marine park, is governed by a set of rules that include prohibitions against fishing and anchoring. Conceivably, these regulations reduce anthropogenic impacts at Raffles Lighthouse and Sisters, and thus we observe no winnowing effect but rather an increase in microbial diversity with increasing host age. The positive age-dependent diversity pattern seen on these two islands is congruent with those observed for microbiomes of humans and other complex organisms^18,19^. It might simply be the case that older corals have had more time to acquire a greater diversity of bacterial types from less-disturbed environments. This coupled with the larger size and developmental changes as a larva grows into an adult colony with complex and varied niches contribute to the higher microbial diversity in older corals^15^.

Senescence has been suggested as a process that reduces bacterial diversity in the microbiome of higher organisms^33–35^. The oldest coral aged in our work came from Kusu and are estimated at 103 year. To put this in context, *Porites* corals have been documented as healthy and still growing at more than 1,000 years old^36^, and 200 year-old *P. lutea* have been recorded in this region^37^. There is even debate on whether corals actually senesce and age at all^38^. We note that *P. lutea* older than 103 year have not been examined here as they are not known to exist in Singapore, so the full effects of senescence on bacterial diversity is not tested. Therefore, whether senescence can explain the reduction in observed bacterial diversity with increasing host age of corals at Jong and Kusu remains uncertain. Furthermore, our results show that older corals collected from Raffles Lighthouse and Sisters tend to harbour the most diverse bacterial communities, suggesting that either corals do not undergo senescence, that the corals we analysed are too young to be senescing, or that senescence does not affect the coral microbiome in the same way as seen in other animal hosts. Additional work could be performed in regions where much larger and consequently older *P. lutea* corals are found in order to investigate the effects of senescence on host-associated microbial diversity.

Like other studies examining microbial diversity and coral-associated bacteria throughout Singapore and the region, we show significant differences in microbial community structure among sampling locations^10,24,39,40^. All samples are generally dominated by Proteobacteria and Cyanobacteria, and samples collected from Tanah Merah appear most distinct in comparison to other sites. Tanah Merah is the easternmost location and is closest to the mainland; the land immediately adjacent to the study site has been undergoing extensive development and coastal reclamation^31^. These major disturbances may be a contributing factor to the coral-associated bacterial community structure found here, especially since similar community responses and differences driven by coastal change have been reported in marine environments^8^. Salinity fluctuations are also much more likely at the Tanah Merah site—a consequence of its proximity to the mainland where freshwater runoff is more prevalent—and these can drive changes in bacterial communities associated with corals^41^. Such fluctuations are much less likely at the other locations where the environment is reported to be homogeneous^10,42,43^.

The high prevalence of Proteobacteria in all samples is in agreement with findings from the Gulf of Thailand and Andaman Sea on the same species^24^. However, we report a much higher occurrence of Cyanobacteria in Singapore, this bacterial phylum is approximately five times more abundant in comparison to those sampled from Thailand^24^. Cyanobacteria, while an essential component of functioning coral reefs, can be an indicator of degraded marine ecosystems at high abundances^44,45^. The much higher prevalence of Cyanobacteria associated with *P. lutea* in Singapore when compared to other Southeast Asian regions is likely indicative of the many pressures faced by the coral reef environment of Singapore (e.g., proximity to a large urban population, major global shipping and petrochemical processing hub etc.).

Despite these obvious pressures, the coral reefs of Singapore continue to persist and support nearly 200 species each of corals and fishes within the 13 km^2^ of coral reef habitat in Singapore^46^. The marine environment of Singapore provides a unique and important opportunity to study coral reefs that are in close proximity to a dense and highly urbanised human population. As the world’s population undergoes rapid urbanisation and mass migration towards coastal areas occurs^47^, the present-day reefs of Singapore may prove useful and relevant as case studies for the reefs of the near future.

The work we perform here, based on the direct measurements of coral age rather than crude estimation by proxy, shows that host age is not a consistent predictor of coral-associated microbial diversity or richness, at least in *Porites lutea*. Host-associated microbial diversity and richness can even vary considerably between colonies of the same age, and communities are structured significantly by location but not coral age. We do not conclude this generally for the relationship between coral age and microbial diversity, but the results are based on accurate rather than crudely estimated measurements of coral age. Our analysis of differential bacterial abundance shows that three families (and two genera) have significantly different associations with corals in varying age classes, although these associations are not specific to older or younger age classes. In other words, a certain bacterial family is not more likely to associate preferentially with, for example, older corals. None of the collected corals showed any visible or obvious signs of degraded health, but had we collected corals with signs of disease, bleaching or algal overgrowth, we may be able to find bacteria that were associated with these degraded or stressed conditions.

More broadly, our study reveals multiple factors that can affect bacterial diversity and underscores the needs to perform controlled experiments aimed at understanding the mechanisms underlying these drivers of microbial diversity.

## Methods

### Field sampling

#### Bacterial sample collection

A total of 160 *Porites lutea* colonies were sampled from eight reef sites in Singapore (Table 1, Supplementary Fig. S8). All samples were collected at depths between 2 and 4 m, and all appeared healthy, showing no visible signs of bleaching, disease or algal overgrowth. An approximately 3 cm^2^ tissue fragment was removed from each colony. Following Wainwright et al.^10^, tissues were placed in separate sealed containers, immersed in water and placed in the shade until further processing. Samples were preserved in 100% molecular grade ethanol within six hours of collection and stored at −80 °C^48–50^. Species identification of each colony was confirmed via a combination of in situ colony and corallite photographs, as well as observations of the ventral triplet septal arrangement performed under a stereo microscope according to Veron^51^ and Forsman et al.^52^. Analyses indicated that all collected specimens were of one species putatively identified as *Porites lutea*^53,54^.

**Table 1:** Details of coral sampling date, sample sizes and GPS coordinates for each location.

Site	Date collected	Sample size	Samples aged	GPS coordinates	
Hantu	20-Sep-17	20	0	1.227835° N	103.746328° E
Jong	20-Sep-17	20	20	1.215304° N	103.785751° E
Kusu	29-Aug-17	20	11	1.224963° N	103.861880° E
Raffles lighthouse	31-Aug-17	20	20	1.160165° N	103.740344° E
Semakau	11-Oct-17	20	20	1.200136° N	103.755956° E
Sisters	15-Nov-17	20	20	1.212573° N	103.836138° E
Sultan Shoal	14-Feb-18	20	0	1.239384° N	103.648806° E
Tanah Merah	20-Jun-17	20	0	1.312095° N	103.986767° E

#### Coral coring and aging

Of the 160 colonies sampled for bacterial analysis, 91 were cored following fragment collection to determine colony age. Cores were taken with a 3-cm diameter core drill bit using a Nemo Divers edition submersible drill (Oxfordshire, UK). Logistical reasons prevented the collection of cores from all 160 colonies. All cores were drilled along the main growth axis of the coral, and age was determined using standard methodology^23^. Briefly, 7-mm thick longitudinal slices were taken from each core to resolve growth chronology, and linear extension rates were established from annual skeletal banding patterns visualised under ultraviolet light. Measurements were cross-validated with alizarin staining over a 2-year period (see Table 1, Supplementary Table S7 for details of aged corals).

### DNA extraction and 16S rRNA gene community profiling

Genomic DNA was extracted with the *ab*Genix automated nucleic acid extraction system (AITbiotech, Singapore) following the manufacturer’s animal tissue DNA extraction protocol. PCR amplification of the 16S rRNA gene V4 region was performed with the 515F and 806R primers (515F: 5′-GTG CCA GCM GCC GCG GTA A-3′; 806R: 5′-GGA CTA CHV GGG TWT CTA AT-3′). Primers were modified to include Illumina adaptors, a linker and unique barcode^55^. We included peptide nucleic acids (PNAs) to prevent preferential plastid and mitochondrial amplification (mPNA: GGC AAG TGT TCT TCG GA; pPNA: GGC TCA ACC CTG GAC AG) (PNAGENE, Daejeon, South Korea)^56^. Each reaction was performed in a total volume of 25 µl, containing 1 µl of undiluted template, 0.1 µl of KAPA 3G Enzyme (Kapa Biosystems, Inc, Wilmington, MA, USA), 0.75 µl of each primer at 10 µM, 2.5 µl of mPNA and 2.5 µl pPNA at 50 µM, 1.5 µl of 1.5 mg/ml BSA, 12.5 µl KAPA PCR buffer and 3.4 µl of water. PCR cycling protocol was 94 °C for 180 s, followed by 35 cycles of 94 °C for 45 s, 75 °C for 10 s, 50 °C for 60 s and 72 °C for 90 s, with a final extension at 72 °C for 10 min. Negative extraction and PCR controls were included to identify possible contamination issues.

PCR products were visualised on a 1% TBE buffer agarose gel. Normalisation and cleaning of PCR products were performed in SequalPrep normalisation plates (Invitrogen, Frederick, Maryland, USA) and submitted for sequencing on the Illumina MiSeq platform (600 cycles, V3 chemistry, 300-bp paired-end reads) with a 15% PhiX spike (Macrogen, Inc).

### Bioinformatics, sequence processing and analysis

Our bioinformatics workflow involved adaptor removal, quality filtering and trimming, error correction, inference of amplicon sequence variants (ASVs), removal of chimeras, and taxonomic assignment.

First, barcodes and adaptors were removed from demultiplexed sequences with Cutadapt^57^. Reads were filtered based on quality scores and trimmed using the DADA2 package version 1.9.0^58^ in R version 3.4.1 (R Core Team 2013). Forward reads were truncated at 260 bp, and reverse reads at 200 bp. Both forward and reverse reads were filtered based on a max EE (expected error) of 2, and reads were additionally truncated at the end of ‘a good quality sequence’ with the parameter truncQ = 2 (see https://benjjneb.github.io/dada2/ for a detailed explanation of filtering parameters). We then estimated error rates from all quality-filtered reads and merged forward and reverse reads to obtain ASVs. Chimeras were removed with de novo detection in the DADA2 package. Sequenced extraction negatives were used to identify possible contaminants using the prevalence method applied within the *decontam* R package^59^. Remaining ASVs were assigned taxonomy with the RDP classifier^60^ against a training set based on the Silva v132 16S database^61^. ASVs assigned to mitochondrial or chloroplast genomes were removed.

Rarefaction curves were produced using the rarecurve() function implemented in the *vegan* R package version 2.5–2^62^. Raw sequence counts were then converted to relative abundance data. Shannon diversity and richness for each sample were calculated, and non-metric multi-dimensional scaling (NMDS) was performed on the Bray–Curtis dissimilarity matrix of samples using the *phyloseq* R package version 1.25.2^63^. Permutational multivariate analysis of variance (Supplementary Table S5) was performed on the ASV table in two ways, one for all 160 corals and the other only on the 91 corals that were aged, using the adonis() function of the *vegan* R package version 2.5–2^62^. Generalised linear models (GLMs) using coral age, location and their interaction as predictors of ASV richness and Shannon diversity were performed, and the predictor lines for each were fitted on Fig. 1.

Differential bacterial abundance and dispersion of taxa at all ranks were assessed using the *corncob* R package^64^, which used a beta-binomial regression to quantify relative abundance and overdispersion simultaneously. This technique can detect increased variability and decreased taxon stability (or dysbiosis) in host-associated bacteria, and can accommodate taxon absences and high variability in relative abundances. We built our models using coral age class and location as separate predictors of both abundance and dispersion with a Wald test and a false discovery threshold of 0.05.

## Supplementary information

Supplementary Information

## Data Availability

All sequences associated with this work have been deposited at the National Center for Biotechnology Information under BioProject ID: PRJNA563869. The code for reproducing the analyses can be found at https://github.com/gzahn/Porites_Coral_Ages, and is archived at https://doi.org/10.5281/zenodo.3827957.
